# Evolution and extensive reassortment of H5 influenza viruses isolated from wild birds in China over the past decade

**DOI:** 10.1080/22221751.2020.1797542

**Published:** 2020-08-05

**Authors:** Yanfang Cui, Yulei Li, Minghui Li, Lu Zhao, Deli Wang, Jingman Tian, Xiaoli Bai, Yanpeng Ci, Shanshan Wu, Fei Wang, Xiaomei Chen, Shujie Ma, Zhiyuan Qu, Cen Yang, Liling Liu, Jianzhong Shi, Yuntao Guan, Xianying Zeng, Guobin Tian, Pengfei Cui, Guohua Deng, Yongping Jiang, Pucheng Chen, Jinxiong Liu, Xiurong Wang, Hongmei Bao, Li Jiang, Yasuo Suzuki, Chengjun Li, Yanbing Li, Hualan Chen

**Affiliations:** a State Key Laboratory of Veterinary Biotechnology, Harbin Veterinary Research Institute, CAAS, Harbin, People’s Republic of China; b School of Pharmaceutical Sciences, University of Shizuoka, Shizuoka, Japan; c College of Life and Health Sciences, Chubu University, Aichi, Japan

**Keywords:** Influenza A virus, H5 subtype, wild birds, reassortant, virulence

## Abstract

Lethal infection of wild birds with different subtypes of H5 viruses continuously occur. To investigate the genetic evolution and pathogenicity of H5 viruses in wild birds, we performed a detailed genetic and biologic analysis of 27 viruses, including H5N1, H5N2, H5N6, and H5N8 subtypes, that were responsible for avian influenza outbreaks in wild birds in China over the past decade. We found that these 27 viruses, bearing different clades/subclades of HA, were complicated reassortants and formed 12 different genotypes. Ten of the viruses tested were highly pathogenic in chickens, but showed distinct pathotypes in ducks and mice. Five of these 10 viruses, which were all from clade2.3.4.4, could bind human-type receptors. Our findings reveal the diversity of the genetic and biologic properties of H5 viruses circulating in wild birds and highlight the need to carefully monitor and evaluate the risks these viruses pose to animal and public health.

## Introduction

Influenza A viruses are widely distributed among wild birds, which are considered natural reservoirs for avian influenza viruses. Indeed, each of the known hemagglutinin (HA) and neuraminidase (NA) subtypes (H1–H16 and N1–N9, respectively) have been isolated from migratory waterfowl. Migratory birds can rapidly disseminate avian influenza viruses along their flyways. Outbreaks of H5 highly pathogenic avian influenza in poultry have resulted in enormous economic losses to the poultry industry, and their sporadic transmission to humans in several countries has highlighted their potential threat to public health [1–3]. To date, the HA genes of H5 highly pathogenic viruses have evolved into multiple clades, defined by the WHO/OIE/FAO as clades 0–9, with some of these clades further divided into as many as 4-digit subclades [4].

H5N1 virus in wild birds was first reported in 2002 in Hong Kong. In 2005, an H5N1 virus belonging to clade 2.2 led to the first outbreak in wild birds in Qinghai Lake, China, which was the first report of fatal cases in wild birds in the history of influenza [5]. With the migration of wild birds, the virus spread from Asia through Siberia to Europe, the Middle East, and Africa within a few months, and the clade 2.2 of H5N1 is still prevalent in Egypt [6]. In 2009, a new HA clade of H5N1 virus, namely clade 2.3.2, emerged among the wild birds in Qinghai Lake, killing 3000 of them [7]; this virus continues to circulate among populations of wild birds and terrestrial poultry [8].

At the beginning of 2014, a novel subtype of highly pathogenic virus, the H5N8 virus of clade 2.3.4.4, emerged in wild birds in Korea. Migratory birds were suspected to have played a key role in the introduction of clade 2.3.4.4 of H5N8 virus from Southeast Asia to Europe and Africa. The novel highly pathogenic Eurasian lineage of H5N8 influenza A virus was introduced to the Americas via migratory waterfowl flying over the Pacific [9,10]. H5N6 highly pathogenic viruses have also been detected in wild birds in 2014 [11], and have caused 26 human infections in China since May 2014 [12].

In this study, we aimed to investigate the genetic evolution, reassortment, and virulence of H5 viruses detected in wild birds since 2010. Our data highlight the fact that H5 highly pathogenic influenza viruses are actively evolving among wild birds and therefore continue to pose a threat to animal and human health.

## Materials and methods

### Ethical approval

All experiments using animals were carried out in strict accordance with the recommendations in the Guide for the Care and Use of Laboratory Animals of the Ministry of Science and Technology of the People’s Republic of China. All protocols were approved by the Committee on the Ethics of Animal Experiments of the Harbin Veterinary Research Institute (HVRI) of the Chinese Academy of Agricultural Sciences (CAAS).

### Biosafety statement and facility

The diagnosis and all experiments with live H5 viruses were conducted in the enhanced animal biosafety level 3 (ABSL3+) facility in the HVRI of the CAAS, which is approved for such use by the Ministry of Agriculture and Rural Affairs of China.

### Eggs

Embryonated chicken eggs were obtained from Harbin Weike Biotechnology Development Company, Harbin,China.

### Mice

Five-week-old female BALB/c mice were purchased from Vital River Laboratories (Beijing, China).

### Chickens

Four-week-old specific-pathogen-free (SPF) chickens (White Leghorn) were obtained from the Experimental Animal Division of HVRI.

### Ducks

Three-week-old SPF ducks (Shaoxin shelduck, a local bred) were obtained from the Experimental Animal Division of HVRI.

### Virus isolation and identification

The 27 H5 viruses used in this study were isolated from samples of dead wild birds sent to the National Avian Influenza Reference Laboratory for the diagnosis of suspected cases of influenza A virus infection between 2010 and 2017 (Table 1). Identification of the HA subtype in the allantoic fluid of HA-positive chicken eggs was performed by using the hemagglutinin inhibition (HI) test with a panel of H1–H16 reference sera, whereas the NA subtype was verified by RT–PCR analysis using a panel of N1–N9 subtype primers.

### Phylogenetic analysis

Total RNA of influenza A viruses was extracted from the allantoic fluid of virus-infected chicken eggs by using the QIAmp viral RNA mini kit (Qiagen, Hilden, Germany). Reverse transcription PCR was performed by using a panel of gene-specific primers, and products were sequenced by using an Applied Biosystems DNA analyzer (primer sequences can be provided upon request). The nucleotide sequences were edited using the Seqman module of the DNAStar package. Phylogenetic analysis was performed by employing the maximum likelihood method using the Mega 6.0.6 ClustalW software package. The tree topology was evaluated by 1000 bootstrap analyses, and a 97% sequence identity cut-off was used to categorize the groups of each gene segment in the phylogenetic trees. A Bayesian time-resolved phylogenetic tree was also created by using BEAST1.8.4 [13]. Data were evaluated by using the Akaike information criterion in Tracer1.6.

### Receptor binding analysis

Receptor binding specificity was analyzed by using a solid-phase binding assay with two different glycopolymers: α-2,3-siaylglycopolymer [Neu5Acα2-3Galβ1-4GlcNAcβ1-pAP (para-aminophenyl)-alpha-polyglutamic acid (α-PGA)] and α-2,6-sialylglycopolymer [Neu5Acα2-6Galβ1-4GlcNAcβ1-pAP (para-aminophenyl)-alpha-polyglutamic acid (α-PGA)], as previously described [14,15]. For the 10 H5 viruses selected in this study, chicken antiserum against the homologous or same clade viruses was used as the primary antibody. A horseradish peroxidase (HRP)-conjugated goat-anti-chicken antibody (Sigma-Aldrich, St. Louis, MO, USA) was used as the secondary antibody. Two viruses, A/chicken/Hebei/3/2013(H5N2) [CK/HeB/3/13(H5N2)] and A/Sichuan/1/09(H1N1) [Sichuan/1/09(H1N1)], that bind exclusively to 2,3- and 2,6-linked sialic acid (SA) receptors, respectively, were used as controls.

### Intravenous pathogenicity index (IVPI) test

The IVPI of the virus in chickens was determined according to the recommendations of the Office International des Epizooties [16].

### Duck test

Groups of eight 3-week-old SPF ducks were intranasally inoculated with 10^6^ 50% egg infectious dose (EID_50_) of test virus in a volume of 0.1 mL. Three ducks from each group were euthanized on day 3 post-infection (p.i.), and their organs (brain, spleen, Kidneys, pancreas, cecal tonsil, bursa of fabricius, thymus, lung, and larynx) were collected for viral titration in eggs. The remaining five ducks in each group were observed for two weeks for clinical symptoms, as well as for seroconversion. Oropharyngeal and cloacal swabs from all surviving ducks were collected on days 3, 5, 7, and 9 for detection of viral shedding.

### Mouse test

To test the mouse lethal dose (MLD_50_), groups of five 6-week-old female BALB/c mice were intranasally inoculated with 10^1.0^ to 10^6.0^ EID_50_ of test virus in a volume of 50 µL. The control group (5 mice) was mock-infected with phosphate-buffered saline. Mice were monitored daily for weight loss and mortality for 14 days. To evaluate viral replication, groups of three mice were intranasally inoculated with 10^6.0^ EID_50_ of the test virus in a volume of 50 µL, and on day 3 p.i. were euthanized to assess viral titres in their nasal turbinates, lungs, brains, kidneys, and spleens.

## Results

### Different H5 virus subtypes caused disease outbreaks in wild birds in China

**Figure 1.**
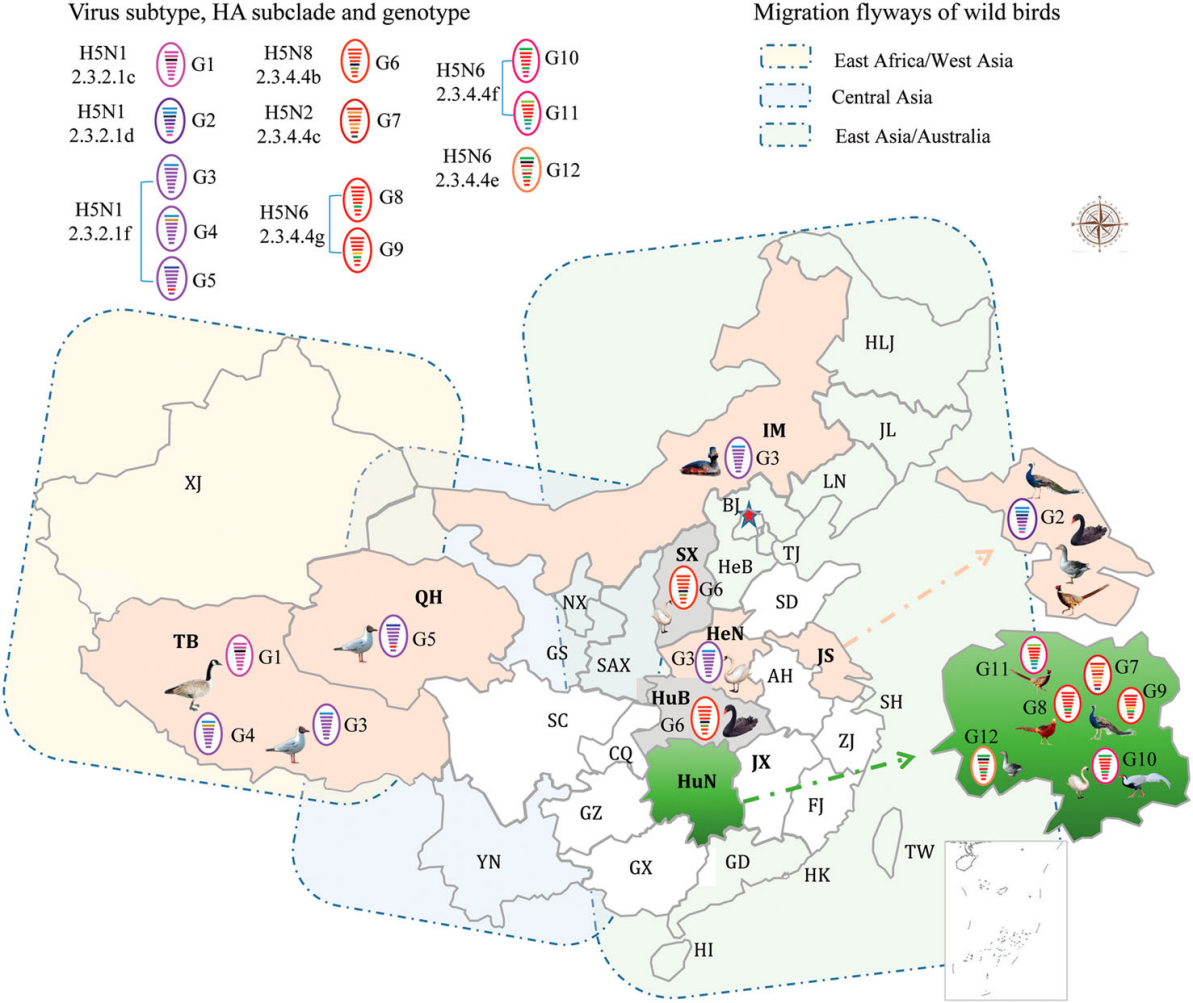
Geographical locations and bird species involved in the wild bird outbreaks caused by different genotypes of H5 viruses.

Between 2010 and 2019, there were 11 outbreaks of H5 avian influenza in wild birds in China. In total, 6,568 wild birds died during these outbreaks. Detailed information about the bird species and the number of birds affected in each outbreak are listed in Table 1. We isolated 27 viruses from the dead birds that were submitted to our laboratory for diagnosis during these outbreaks; these strains included 12 H5N1 strains, one H5N2 strain, 10 H5N6 strains, and four H5N8 strains (Table 1). The H5N1 viruses were detected from wild birds in Tibet, Henan, Inner Mongolia, Jiangsu, and Qinghai, whereas the H5N2 and H5N6 viruses were only detected in the wild birds in Hunan, and the H5N8 viruses were detected in wild birds in Shanxi and Hubei (Table 1, Figure 1).

**Table 1.** H5 avian influenza outbreaks in wild birds in China.

Time	Province	No. of bird died	Bird species/order	Virus information		
				Name	HA clade	IVPI
May 29, 2010	Tibet (TB)	170	brown-headed gull (BHGL)/charadriiformes (CHA)	BHGL/TB/1/2010(H5N1)	2.3.2.1c	/*
				BHGL/TB/2/2010(H5N1)	2.3.2.1c	/
January 5, 2015	Henan (HeN)	93	whooper swan (WS)/anseriformes (ANS)	WS/HeN/1/2015(H5N1)	2.3.2.1f	2.90
May 27, 2015	Inner Mongolia (IM)	300	black-necked grebe (BNG)/ciconiiformes (CIC)	BNG/IM/2/2015(H5N1)	2.3.2.1f	/
May 28, 2015	TB	415	bar-headed Goose (BHG)/ANS, BHGL/CHA	BHG/TB/3/2015(H5N1)	2.3.2.1f	/
				BHGL/TB/4/2015(H5N1)	2.3.2.1f	/
				BHG/TB/5/2015 (H5N1)	2.3.2.1f	/
June 13, 2015	Jiangsu (JS)	1858	black swan (BS)/ANS, greylag goose (GG)/ANS, green peacock (GP)/galliformes (GAL), common pheasant (CP)/GAL	BS/JS/5/2015(H5N1)	2.3.2.1d	/
				GG/JS/6/2015(H5N1)	2.3.2.1d	3.00
				GP/JS/7/2015(H5N1)	2.3.2.1d	/
				CP/JS/8/2015(H5N1)	2.3.2.1d	/
June 16, 2015	Qinghai (QH)	2361	great black-headed gull (GBHG)/CHA	GBHG/QH/9/2015(H5N1)	2.3.2.1f	2.68
December 3, 2015	Hunan (HuN)	381	golden pheasant (GDP)/GAL, CP/GAL	GDP/HuN/10/2015(H5N6)	2.3.4.4g	/
				CP/HuN/11/2015(H5N6)	2.3.4.4g	2.90
				GP/HuN/12/2015(H5N6)	2.3.4.4g	/
				GP/HuN/13/2015(H5N6)	2.3.4.4g	2.76
				GP/HuN/15/2015(H5N6)	2.3.4.4g	/
				GP/HuN/14/2015(H5N2)	2.3.4.4c	2.54
January 27, 2016	HuN	91	GP/GAL silver pheasant (SP)/GAL, WS/ANS	GP/HuN/1/2016(H5N6)	2.3.4.4f	2.68
				CP/HuN/2/2016(H5N6)	2.3.4.4f	2.74
				SP/HuN/3/2016(H5N6)	2.3.4.4f	/
				WS/HuN/4/2016(H5N6)	2.3.4.4f	/
December 24, 2016	Shanxi (SX)	18	WS/ANS	WS/SX/6/2016(H5N8)	2.3.4.4b	2.56
				WS/SX/7/2016(H5N8)	2.3.4.4b	/
January 3, 2017	HuN	1054	GG/ANS	GG/HuN/1/2017(H5N6)	2.3.4.4e	2.62
January 12, 2017	Hubei (HuB)	99	BS/ANS	BS/HuB/2/2017(H5N8)	2.3.4.4b	/
				BS/HuB/3/2017(H5N8)	2.3.4.4b	/

*:/, not done.

### The HA genes of the wild bird viruses belong to two major clades and multiple subclades

The nucleotide sequences analyzed in this study were deposited in the Global Initiative on Sharing Avian Influenza Data database (http://www.gisaid.org) under accession nos. EPI1639776–EPI1639992.

**Figure 2.**
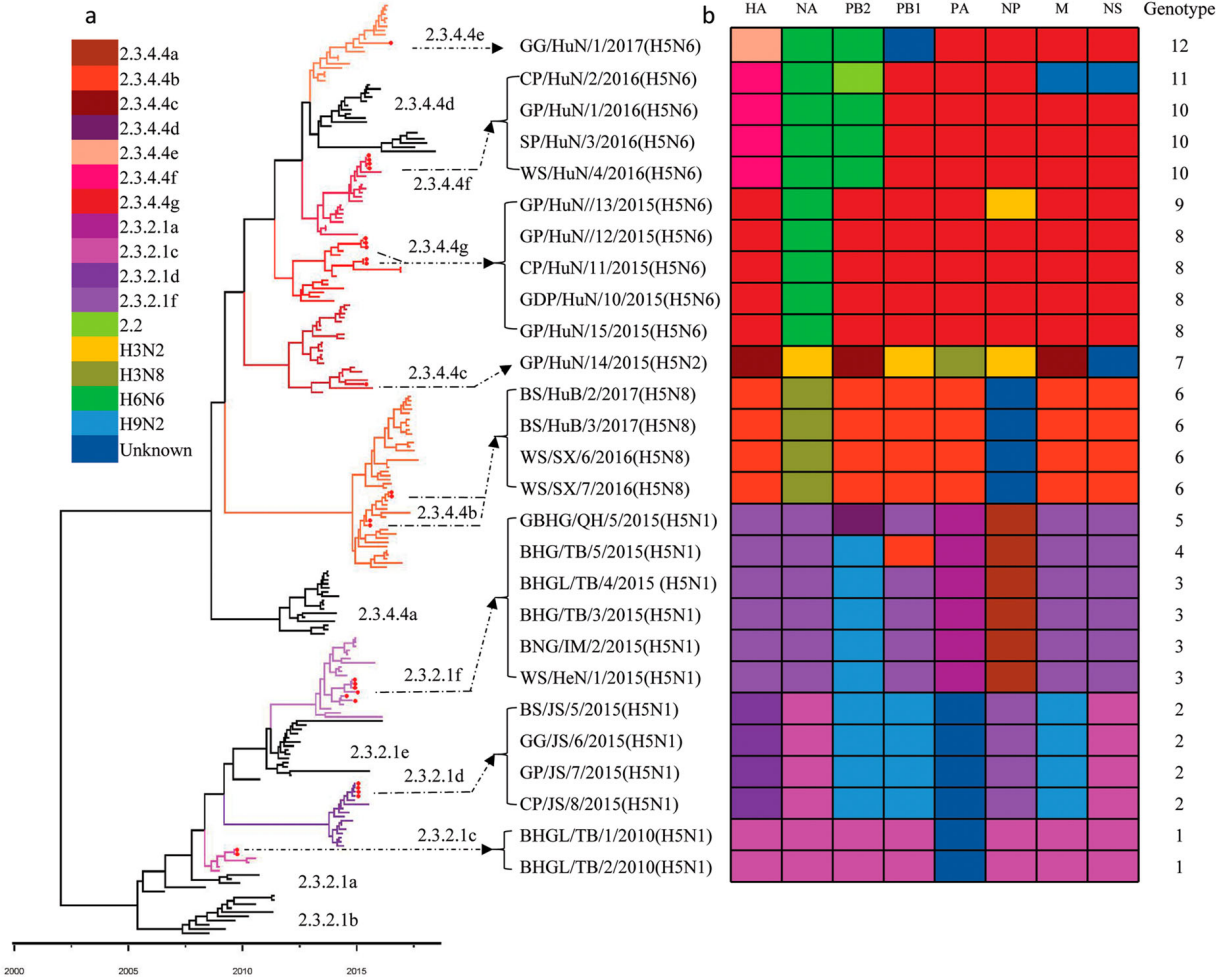
Phylogenetic analysis of H5 wild bird viruses and genotypes. The Maximum Clade Credibility (MCC) tree of the HA gene was constructed by using the package BEAST (v1.8.4). The clades shown in different colours in the tree were annotated by using FigTree (v1.4.4) (a), and the origins of each gene segment are indicated by different coloured bars (b). Phylogenetic trees with complete information are provided as Supporting Figures S1 and S2.

We integrated our evolutionary analysis of the HA gene of 27 viruses onto the same timescale to elucidate the timing and pattern of divergence. As shown in Figure 2(a), and Figure S1A, the HA genes of the 27 viruses belonged to two different evolution clades, namely clade 2.3.2.1 and clade 2.3.4.4. The HA genes of the 12 H5N1 viruses were in clade 2.3.2.1, and located in three different subclades: subclade 2.3.2.1c [BHG/TB/1/2010(H5N1) and BHG/TB/2/2010(H5N1)], subclade 2.3.2.1d [BS/JS/5/2015(H5N1), GG/JS/6/2015(H5N1), GP/JS/7/2015(H5N1) and CP/JS/8/2015(H5N1)], and subclade 2.3.2.1f [WS/HeN/1/2015(H5N1), BNG/IM/2/2015(H5N1), BHG/TB/3/2015 (H5N1), BHGL/TB/4/2015(H5N1), BHG/TB/5/2015(H5N1) and GBHG/QH/9/2015(H5N1)] (Figure 2). The HA genes of the other 15 viruses (one H5N2, 10 H5N6, and four H5N8) were in clade 2.3.4.4 and located in five different subclades: subclade 2.3.4.4b [BS/HuB/2/2017(H5N8), BS/HuB/3/2017(H5N8), WS/SX/6/2016(H5N8), and WS/SX/7/2016(H5N8)], subclade 2.3.2.4c [GP/HuN/14/2015(H5N2)], subclade 2.3.4.4e [GG/HuN/1/2017(H5N6)], subclade 2.3.4.4f [GP/HuN/1/2016(H5N6), CP/HuN/2/2016(H5N6), SP/HuN/3/2016(H5N6) and WS/HuN/4/2016(H5N6)], and subclade 2.3.4.4g [GDP/HuN/10/2015(H5N6), CP/HuN/11/2015(H5N6), GP/HuN/12/2015(H5N6), GP/HuN/13/2015(H5N6), and GP/HuN/15/2015(H5N6)] (Figure 2). Subclade 2.3.4.4a contains most of the H5N6 strains that emerged in China and Vietnam in 2014 and caused poultry outbreaks and human infections; however, none of the H5N6 viruses in this study belonged to this subclade. The subclades 2.3.4.4b and 2.3.4.4c also contained the HA of the H5N8 viruses responsible for the outbreaks in Asia, Europe, and Africa between 2014 and 2018, whereas subclade 2.3.4.4c contained the HA of H5N8 viruses that triggered the avian influenza outbreak in Korea in 2014 and then spread to Europe and North America via the migration of wild birds, and the HA of H5N2 which caused the poultry outbreaks in America in 2014–2015 (Figure 2). In addition to GG/HuN/1/2017(H5N6), subclade 2.3.4.4e also included viruses that circulated in East Asia and caused outbreaks in Korea and Japan during the 2016–2017 winter season (Figure S1a). The HA genes of other viruses in subclades 2.3.4.4f and 2.3.4.4 g were mainly from the previously detected H5N1 and H5N6 strains in China and Vietnam (Figure 2).

### The H5 viruses derived their NA genes from H3N2, H6N6, and H3N8 viruses and formed different subtypes

Phylogenic analysis indicated that the NA gene of influenza viruses formed different lineages (Figure S1b–e). The NA gene of the 12 H5N1 viruses in this study belonged to two different N1 NA lineages (Figure S1b), the N2 NA gene of GP/HuN/14/2015(H5N2) belonged to the H3N2 lineage (Figure S1c), the NA gene of the 10 H5N6 viruses in the study belonged to the H6N6 lineage (Figure S1d), and the NA gene of the four H5N8 viruses belonged to the H3N8 lineage (Figure S1e), indicating that the H5N2, H5N6, and H5N8 viruses may have derived their NA genes from the H3N2, H6N6, and H3N8 viruses, respectively.

### The internal genes of the H5 wild bird viruses are derived from multiple subtypes of avian influenza viruses

The internal genes of the H5 viruses showed clear diversity (Figure S1f–k). Most of the internal genes were clustered with the genes of earlier H5N1 viruses, whereas the PB2 gene of the ten H5N1 viruses (clade 2.3.2.1d and 2.3.2.1f) and that of the H9N2 viruses located in the same lineage, and the PB2 of the H5N6 viruses in clades 2.3.4.4e and 2.3.4.4f located in the same lineage with the PB2 gene of the H6N6 viruses (Figure S1f). The PB1 of GP/HuN/14/2015(H5N2) and GG/HuN/1/2017(H5N6) clustered with the PB1 of different subtypes of low pathogenic influenza viruses (Figure S1g). The PA of GP/HuN/14/2015(H5N2), and the NP of GP/HuN/14/2015(H5N2) and GP/HuN/13/2015(H5N6) located in the same lineages with those of the H3N2 viruses (Figure S1h,i). The M of CP/HuN/2/2016(H5N6) and four H5N1 viruses (subclade 2.3.2.1d) and the NS of CP/HuN/2/2016(H5N6) located in the same lineages with those of the H9N2 viruses (Figure S1j,k).

### The H5 wild bird viruses have formed 12 different genotypes

**Figure 3.**
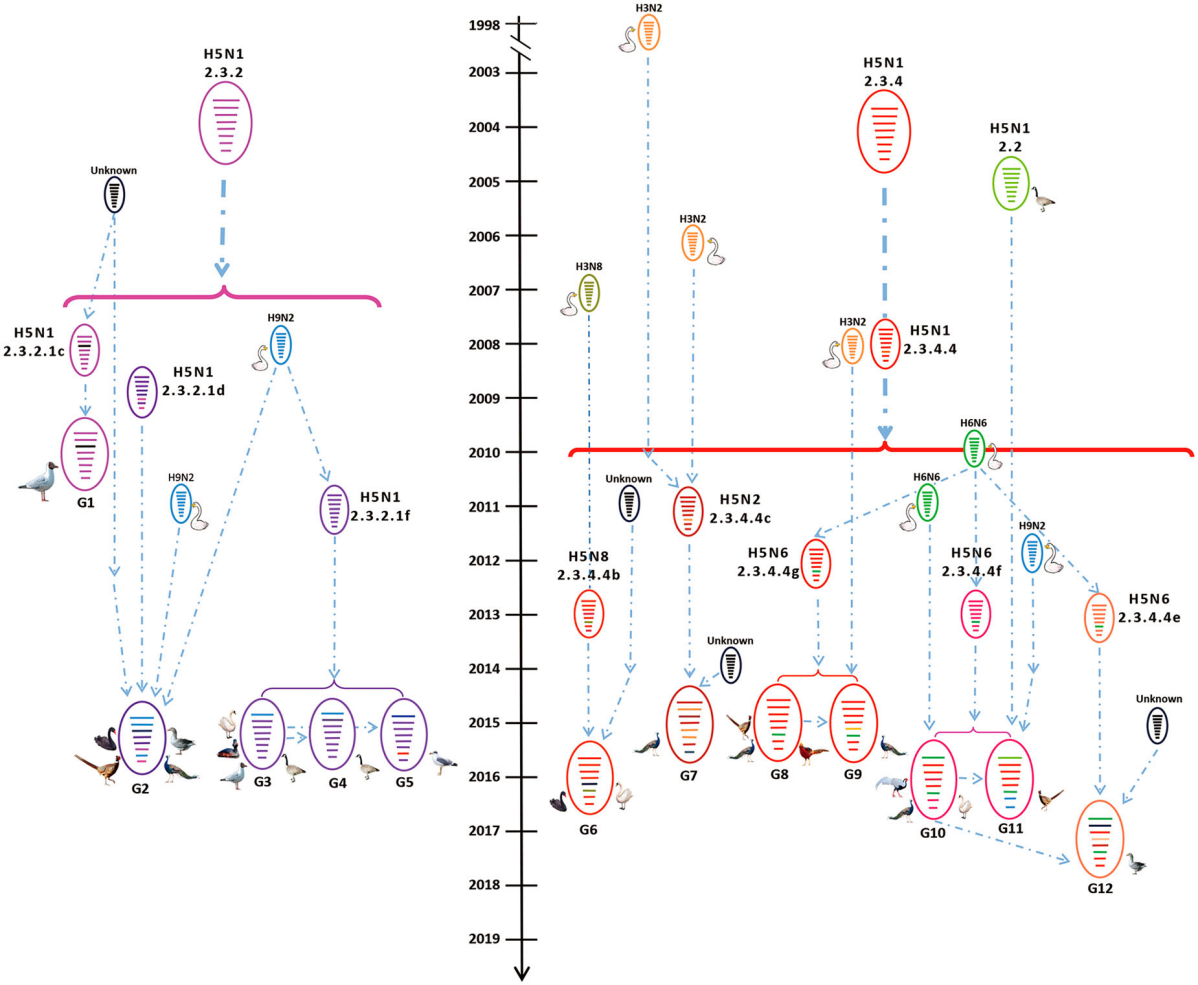
Diagrammatic representation of the formation of different H5 reassortants. Viral particles are represented by coloured ovals containing horizontal bars representing the 8 gene segments (from top to bottom: PB2, PB1, PA, HA, NP, NA, M, and NS). Segments in descendant viruses are coloured according to their corresponding source virus (top) to illustrate gene ancestry through reassortment events. Possible donor viruses are adjacent to arrow tails; arrowheads point to the resulting reassortants. The timeline in the middle indicates the possible time of virus emergence or reassortment events. The tMRCA was estimated by using the Bayesian Markov chain Monte Carlo method in the BEAST v1.8.4 software package.

On the basis of their genomic diversity, the 27 viruses were divided into 12 genotypes (G1–G12); the formation of these genotypes is illustrated in Figure 3 according to the results of our phylogenetic and the time to most recent common ancestor (tMRCA) analyses. The G1–G5 genotypes include H5N1 reassortants bearing different subclades of clade 2.3.2.1 HA and one or more internal gene(s) derived from different H9N2 viruses, clade 2.3.4.4 H5 viruses, or unknown viruses (Figure 3). The four G6 viruses are H5N8 reassortants bearing the HA gene of subclade 2.3.4.4b, the NA gene of H3N8 viruses, and the NP gene of unknown viruses (Figure 3). The G7 virus [GP/HuN/14/2015(H5N2)] is a reassortant bearing the HA, PB2, and M genes of clade 2.3.4.4c H5 viruses, the NA, PB1, and NP genes of H3N2 viruses, the PA of an H3N8 virus, and the NS from an unknown virus (Figure 3). The four G8 viruses are reassortants of clade 2.3.4.4 g H5 viruses bearing the NA gene from H6N6 viruses. Of note, the G9 virus is a reassortant of a G8 virus and an H3N2 virus, which provided the NP gene (Figure 3). The three G10 viruses are reassortants of clade 2.3.4.4f H5 viruses bearing the NA and PB2 of H6N6 viruses (Figure 3). The G11 virus is a reassortant of a G10 virus, an early clade 2.2 virus, which provided the PB2 gene, and an H9N2 virus, which provided the M and NS genes, whereas the G12 virus is a reassortant of a G10 virus that acquired its HA gene from a clade 2.3.4.4e virus and PB1 gene from an unknown virus. These analyses demonstrate that the H5 viruses evolved not only by accumulating mutations in their genome, but also through actively reassorting with other subtypes of influenza viruses.

### Key mutations or deletions in the H5 wild bird viruses

We compared the amino acids of the 27 viruses and found amino acid mutations at eight positions in HA that have previously been reported to increase affinity for human-type receptors in H5 wild bird viruses: 94N, 133A, 156A, 183G/N, 188I/A, 189R/A/D/N/S, 192 K, and 235S (Table 2). Of note, the clade 2.3.2.1 viruses only had three or four of these eight mutations in their HA, whereas the clade 2.3.4.4 viruses had five to eight mutations in their HA. The four H5N6 viruses bearing clade 2.3.4.4f HA had all eight mutations (Table 2).

We found that all of the H5N1 viruses had a deletion of 10 amino acids at positions 49–68 of NA (the stalk of N1 NA), and all of the H5N6 viruses had a deletion of 12 amino acids at positions 59–70 of NA (the stalk of N6 NA), whereas the H5N2 NA and H5N8 NA did not have any deletions in their stalk (Table 2). The PB2 627 K is a very important mutation that can increase the virulence of different avian influenza viruses in mammals [5,17], and this mutation was detected in the GBHG/QH/9/2015(H5N1) and CP/HuN/2/2016(H5N6) viruses (Table 2). All of the 27 viruses had the 42S residue in their NS1, which is important for the virus to antagonize the host antiviral response and for lethality in mice [18]. All of the H5N1 viruses and nine of the 10 H5N6 viruses had a deletion of five amino acids at positions 80–84 of their NS1 gene; this deletion has been reported to increase the virulence of H5 viruses in mice [19]. Moreover, 12 viruses, including 10 H5N1 viruses, one H5N2 virus, and one H5N6 virus, acquired an M2 mutation (27I or 31N) that is known to increase resistance to amantadine and rimantadine (Table 2).

### The h5 wild bird viruses are highly pathogenic in chickens

We selected ten viruses (Table 1) and evaluated their virulence in different animals and their receptor binding properties.

The pathogenicity of the 10 viruses in chickens was determined by testing their IVPI as described previously [2]. The IVPI values of the ten viruses ranged from 2.54–3.0 (Table 1), indicating that all of the tested viruses were highly pathogenic for chickens.

### The H5 wild bird viruses replicate efficiently but exhibit different pathotypes in ducks

Groups of eight ducks were inoculated with the test viruses. Three ducks in each virus-inoculated group were euthanized on day 3 post-inoculation (p.i.) to test for viral replication in their organs; the other five ducks in each group were monitored for two weeks for virus shedding and death. Three uninfected ducks were cohoused to monitor virus transmission. We found that all 10 of the viruses replicated to high titres in all nine organs and tissues tested, including brain, spleen, kidneys, pancreas, cecal tonsil, bursa of fabricius, thymus, lung, and larynx (Table 3, Figure S3). All of the viruses were shed by the inoculated ducks and were transmitted from the inoculated ducks to contact ducks (Table 3). Three viruses were non-lethal and did not kill any ducks, three viruses were highly lethal and killed all of the inoculated ducks, whereas the other four strains killed one or four of the five inoculated ducks (Table 3). These results show that the ten H5 viruses isolated from wild birds can replicate and transmit efficiently in ducks, although their virulence varies among strains (Table 3).

**Table 3.** Replication and virulence of different H5 viruses in ducks.

Virus	Replication in organs (mean titre)^a^		Virus shedding on different days post inoculation, positive/total (mean titre: oropharynx/cloaca)^b^						Death/total		Seroconversion: positive/total (mean HI antibody titre, log2)	
			Day 3		Day 5		Day 7					
	Lung	Brain	Inoculated	Contact	Inoculated	Contact	Inoculated	Contact	Inoculated	Contact	Inoculated	Contact
GG/JS/6/2015(H5N1)	4.8	5.6	/	/	/	/	/	/	5/5	3/3	/	/
WS/HeN/1/2015(H5N1)	5.0	7.2	5/5 (3.7/1.3)	3/3 (2.7/0.9)	5/5 (2.0/1.5)	3/3 (3.0/0.9)	3/3 (1.2/<)	3/3 (1.6/1.1)	4/5	0/3	1/1 (6.0)	3/3 (3.7)
GBHG/QH/9/2015(H5N1)	6.4	3.3	5/5 (3.4/0.9)	3/3 (4.2/2.3)	0/5	3/3 (2.2/0.9)	0/5	0/3	0/5	0/3	5/5 (6.4)	3/3 (5.0)
GP/HuN/14/2015(H5N2)	5.4	4.3	5/5 (3.8/1.9)	3/3 (1.8/1.6)	5/5 (1.3/0.9)	3/3 (1.6/1.5)	0/5	0/3	0/5	0/3	5/5 (6.2)	3/3 (5.3)
GP/HuN/13/2015(H5N6)	4.8	5.0	5/5 (4.0/2.1)	3/3 (3.1/2.8)	5/5 (2.7/2.4)	3/3 (3.4/1.7)	/	0/3	5/5	0/3	/	3/3 (6.0)
CP/HuN/11/2015(H5N6)	5.6	5.3	5/5 (3.8/2.9)	3/3 (4.3/3.9)	5/5 (2.9/1.9)	3/3 (1.0/0.9)	2/4 (1.3/0.9)	0/3	1/5	0/3	4/4 (6.3)	3/3 (6.0)
CP/HuN/2/2016(H5N6)	5.8	4.8	4/4 (4.6/3.2)	3/3 (5.4/2.4)	1/3 (1.2/0.9)	3/3 (4.8/2.7)	2/2 (1.0/0.9)	2/2 (1.2/1.4)	4/5	1/3	1/1 (7.0)	2/2 (7.5)
GP/HuN/1/2016(H5N6)	4.3	5.0	5/5 (4.0/3.0)	3/3 (3.6/2.8)	1/4 (0/3.8)	3/3 (1.2/0.9)	0/4	0/3	1/5	0/3	4/4 (5.3)	3/3 (5.7)
GG/HuN/1/2017(H5N6)	5.8	5.8	5/5 (4.9/3.3)	3/3 (4.8/2.4)	2/5 (4.4/0.9)	3/3 (3.1/1.6)	/	/	5/5	3/3	/	/
WS/SX/6/2016(H5N8)	4.8	5.8	5/5 (4.4/3.3)	3/3 (4.8/2.0)	5/5 (1.9/0.9)	3/3 (2.8/0.9)	0/5	3/3 (1.8/0.9)	0/5	1/3	5/5 (4.8)	2/2 (4.5)

^a^Viral titre of all ten organs tested were show in supporting Figure 3.

^b^Swabs of survival ducks on day 9 p.i. were also collected, virus was only detected in one contact duck of the WS/HeN/1/2015(H5N1)-inoculated group, with titres of 1.0 and 0.9 in the oropharynx and cloaca, respectively. /, all ducks died; <, virus was not detected in the cloacal swabs.

**Table 2.** Mutations detected in the H5 wild bird viruses that contributed to increased binding to human-type receptors, virulence in mammals, and resistance to antiviral drugs (blank cell indicates no such mutation at that position).

Virus	Clade	Amino acids in HA that may increase the affinity to human-type receptor (H5 numbering)								Mutations or deletions in different genes that may increase virulence in mice				Mutations in M2 that increase resistance to amantadine and rimantadine	
										NA^a^	PB2	NS1			
		94N	133A	156A	183N	188 I/A	189R/A/D/N/S	192K	235S	Stalk deletion	627K	42S	80–84 deletion	27I/A	31A/N
BHGL/TB/1/2010(H5N1)	2.3.2.1c	N	A				R			Yes		S	Yes		
BHGL/TB/2/2010(H5N1)	2.3.2.1c	N	A				R			Yes		S	Yes		
WS/HeN/1/2015(H5N1)	2.3.2.1f	N	A							Yes		S	Yes	I	
BNG/IM/2/2015(H5N1)	2.3.2.1f	N	A							Yes		S	Yes	I	
BHG/TB/3/2015(H5N1)	2.3.2.1f	N	A							Yes		S	Yes	I	
BHGL/TB/4/2015(H5N1)	2.3.2.1f	N	A							Yes		S	Yes	I	
BHG/TB/5/2015 (H5N1)	2.3.2.1f	N	A							Yes		S	Yes	I	
BS/JS/5/2015(H5N1)	2.3.2.1d	N	A			I	A			Yes		S	Yes		N
GG/JS/6/2015(H5N1)	2.3.2.1d	N	A			I	A			Yes		S	Yes		N
GP/JS/7/2015(H5N1)	2.3.2.1d	N	A			I	A			Yes		S	Yes		N
CP/JS/8/2015(H5N1)	2.3.2.1d	N	A			I	A			Yes		S	Yes		N
GBHG/QH/9/2015(H5N1)	2.3.2.1f	N	A	A						Yes	K	S	Yes	I	
GDP/HuN/10/2015(H5N6)	2.3.4.4g	N	A	A	N		D	K		Yes		S	Yes		
CP/HuN//11/2015(H5N6)	2.3.4.4g	N	A	A	N		D	K		Yes		S	Yes		
GP/HuN//12/2015(H5N6)	2.3.4.4g	N	A	A	N		D	K		Yes		S	Yes		
GP/HuN/15/2015(H5N6)	2.3.4.4g	N	A	A	N		D	K		Yes		S	Yes		
GP/HuN/13/2015(H5N6)	2.3.4.4g	N	A	A	N		D	K		Yes		S	Yes		
GP/HuN/14/2015(H5N2)	2.3.4.4c		A	A	N	I	N	K		No		S	No		N
GP/HuN/1/2016(H5N6)	2.3.4.4f	N	A	A	N	A	S	K	S	Yes		S	Yes		
SP/HuN/3/2016(H5N6)	2.3.4.4f	N	A	A	N	A	S	K	S	Yes		S	Yes		
WS/HuN/4/2016(H5N6)	2.3.4.4f	N	A	A	N	A	S	K	S	Yes		S	Yes		
CP/HuN/2/2016(H5N6)	2.3.4.4f	N	A	A	N	A	S	K	S	Yes	K	S	No		N
GG/HuN/1/2017(H5N6)	2.3.4.4e	N	A	A	N		N	K		Yes		S	Yes		
WS/SX/6/2016(H5N8)	2.3.4.4b		A	A	N		N	K		No		S	No		
WS/SX/7/2016(H5N8)	2.3.4.4b		A	A	N		N	K		No		S	No		
BS/HuB/2/2017(H5N8)	2.3.4.4b		A	A	N		N	K		No		S	No		
BS/HuB/3/2017(H5N8)	2.3.4.4b		A	A	N		N	K		No		S	No		

^a^H5N1 viruses had a deletion of 10 amino acids at positions 49–68 of NA (the stalk of N1 NA), and all of the H5N6 viruses had a deletion of 12 amino acids at positions 59–70 of NA (the stalk of N6 NA).

### Some H5 wild bird viruses bind to α-2,6-linked SAs

**Figure 4.**
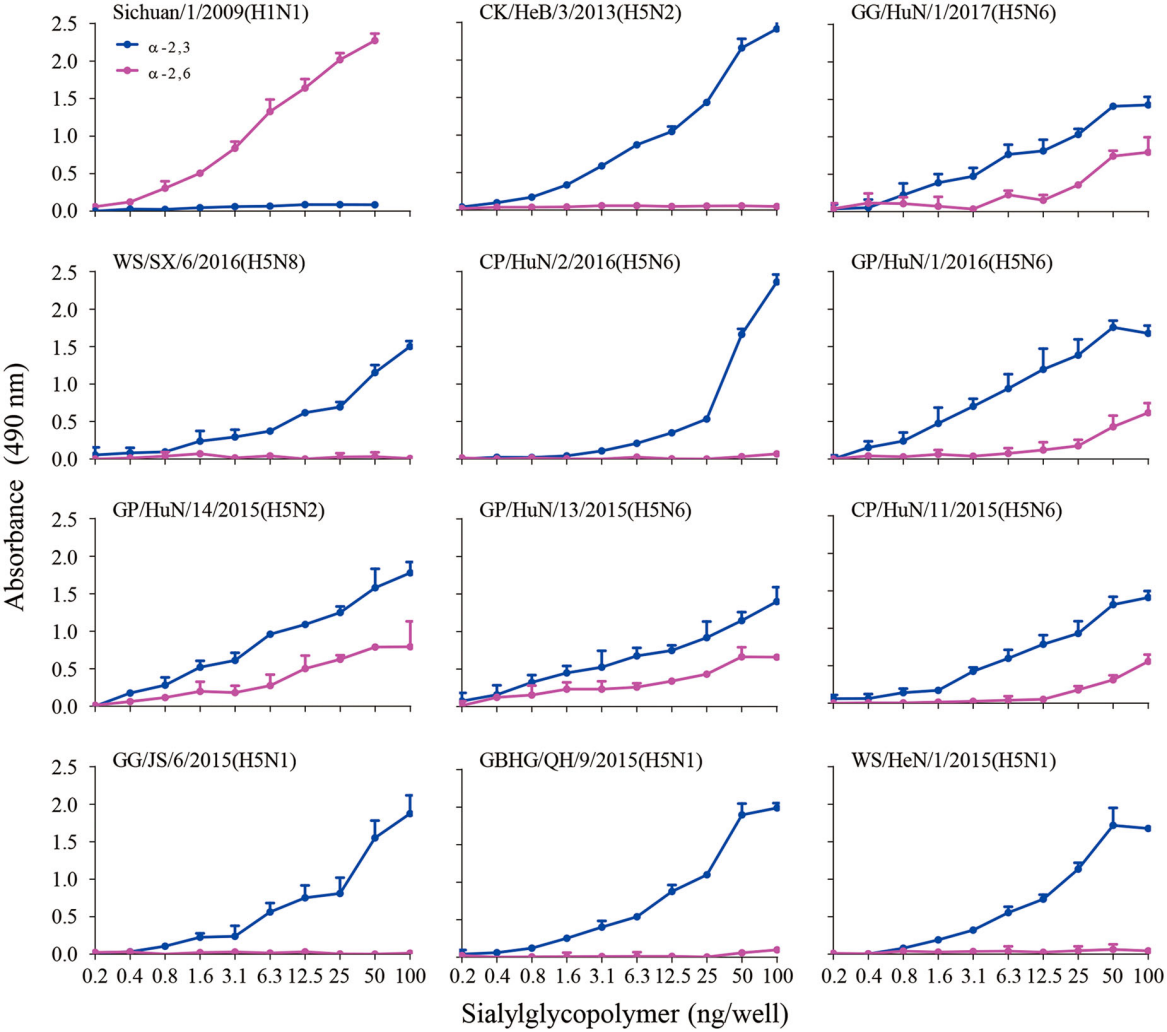
Receptor binding properties of H5 wild bird viruses. Binding properties were analyzed by using two types of receptors, α-2,6-linked SAs (human-type receptors) and α-2,3-linked SAs (avian-type receptors).

To evaluate the receptor-binding properties of the 10 isolates, we assessed their binding to α-2,3-siaylglycopolymer and α-2,6-siaylglycopolymer by using solid-phase binding assays. Similar to the previous reports [15,16], the control viruses CK/HeB/3/2013(H5N2) and Sichuan/1/2009(H1N1) exclusively bound to the avian-type receptors and human-type receptors, respectively (Figure 4). Five of the 10 test viruses exclusively bound to the avian-type receptors, whereas the other five viruses bound to both avian-type receptors and human-type receptors, although their affinity to the human-type receptors was much lower than that to the avian-type receptors (Figure 4). Of note, the viruses that bound to the human-type receptors were all H5N2 or H5N6 viruses. These results demonstrate that the H5 circulating in wild birds have distinct receptor-binding properties and that the viruses of clade2.3.4.4 have acquired the ability to bind to human-type receptors.

### The H5 wild bird viruses exhibit different virulence in mice

**Figure 5.**
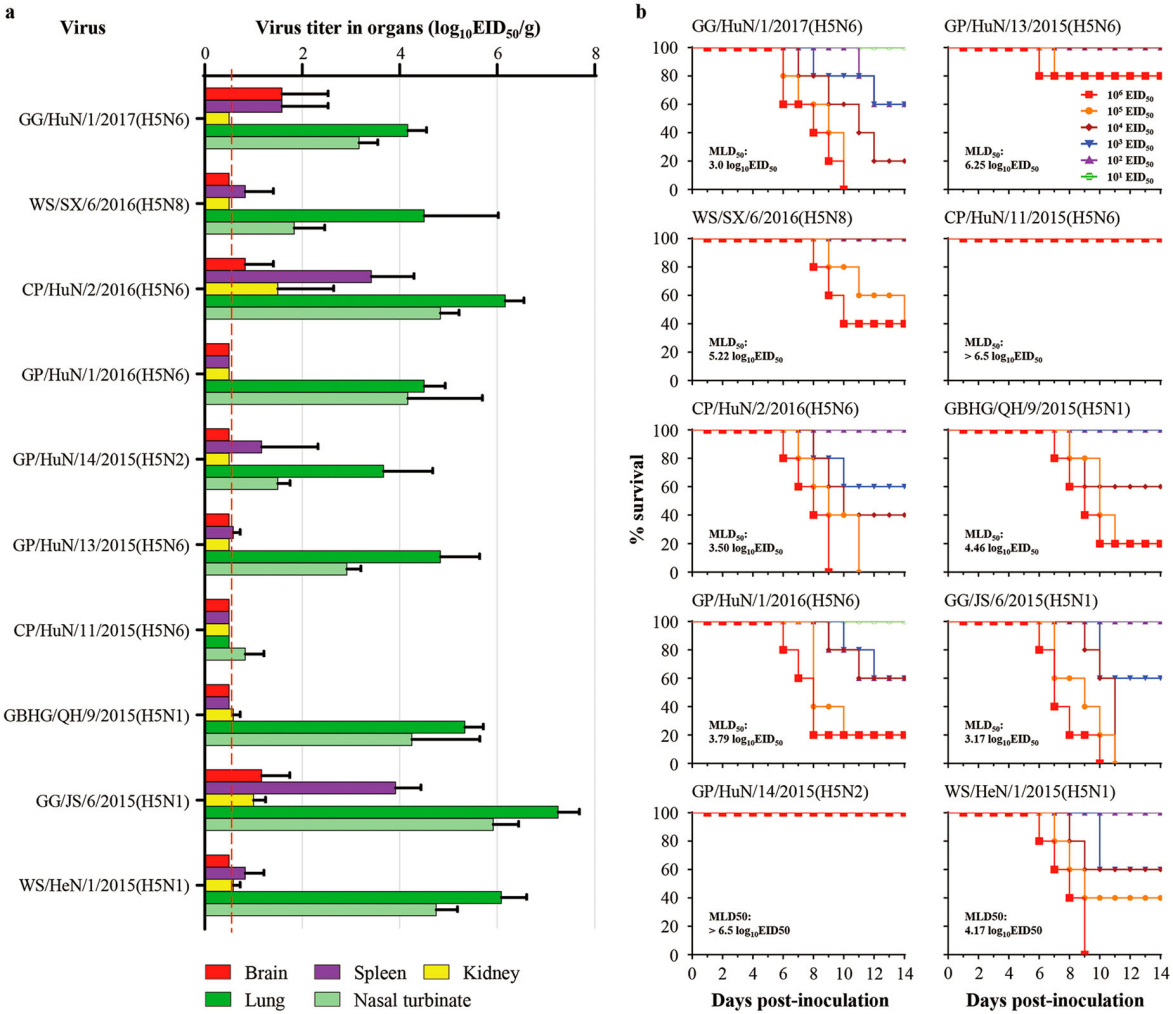
Replication and virulence of H5 wild bird viruses in mice. (a) Viral titres in organs of mice that were euthanized on day 3 post-inoculation with 10^6^EID_50_ of the test virus. (b) MLD_50_ of each test virus.

The replication of the ten viruses in the organs of mice is shown in Figure 5. CP/HuN/11/2015(H5N6) replicated in only the nasal turbinate of mice, whereas the other nine viruses replicated in both the nasal turbinates and lungs of mice, and some of these viruses were also detected in the brain, spleen, or kidneys of mice (Figure 5(a)). The virulence of the 10 viruses in mice varied widely from low lethality to highly lethal. Three viruses, GG/HuN/1/2017(H5N6), CP/HuN/2/2016(H5N6), and GG/JS/6/2015(H5N1), were highly lethal in mice, with MLD_50_ values of less than 3.5 log EID_50_; five viruses – GP/HuN/1/2016(H5N6), GBHG/QH/9/2015(H5N6), WS/HeN/1/2015(H5N1), WS/SX/6/2016(H5N8), and GP/HuN/13/2015(H5N6) – showed medium pathogenicity in mice, with MLD_50_ values ranging from 3.5 to 6.5 log_10_EID_50_; and two viruses, GP/HuN/14/2015(H5N2) and CP/HuN/11/2015(H5N6), were of low lethality in mice, in that they did not kill any mice even at the dose of 10^6^EID_50_, with an MLD_50_ value of >6.5 log_10_ EID_50_ (Figure 5(b)). These results show that the H5 wild bird viruses have different replication and pathotypes in mice. There were no correlations between the pathotypes and the subtypes or genotypes, suggesting that the individual mutations, rather than the genome constellation, may determine their pathogenic potential.

## Discussion

Our study of 27 H5 wild bird viruses revealed that H5N1, H5N2, H5N6, and H5N8 viruses bearing different clades/subclades of the H5 HA gene were responsible for the wild bird influenza outbreaks in China over the last decade. These viruses formed multiple genotypes by accumulating mutations in their HA gene, acquiring the NA gene from H3N2, H4N6, or H3N8 viruses and internal genes from H9N2 and other unknown viruses. Moreover, ten of the tested viruses were highly pathogenic in chickens, but had different pathotypes in ducks and mice, and five of these ten viruses tested bound to human-type receptors.

Gene fragment reassortment is a major mechanism for the evolution of influenza virus [16]. Previous studies have reported that a predominated genotype of H9N2 influenza virus circulating in poultry in China provided the entire six internal genes to the H7N9, H10N8, and H5N6 viruses and facilitated human infections with these strains [14,20,21]. However, this kind of reassortment did not occur in the H5 wild bird viruses, as evidenced by the fact that the wild bird H5N1 viruses frequently derived their NA gene and only one or two internal genes from H3N2, H3N8, H6N6, H9N2, and other low pathogenic avian influenza viruses detected in ducks and wild birds.

The virulence of influenza virus is a polygenic trait, and the PB2 627 K residue is important for influenza virus virulence in mammals [5,15,22]. The GBHG/QH/9/2015(H5N1) virus had 627 K in its PB2, yet its virulence in mice was intermediate. We speculate that some other residues in its genome may have limited its virulence in mice, as observed in the H7N9 virus by Ma et al [23]. The NA stalk and NS deletions (Table 2) have been reported to increase the virulence of H5N1 viruses in mice [19]. Our present study and a previous study by others suggest that H5N2 and H5N8 viruses are not highly lethal to mammals [22], and that their compromised virulence may be partially attributable to their NA and NS genes, which do not have the virulence-increasing deletions (Table 2).

Receptor-binding specificity is a key factor for host adaptation, and binding to human-type receptor may facilitate the replication and transmission of avian influenza virus in humans [24]. Five of the viruses we tested (one H5N2 and four H5N6) had dual receptor-binding properties. Although their affinity for the human-type receptors was lower than that for the avian-type receptor, a few more mutations may dramatically increase this affinity for the human-type receptor. Therefore, it is important to continue evaluating the receptor-binding properties of H5N2 and H5N6 viruses circulating in nature.

The pathogenicity of avian influenza virus to ducks has previously been reported to vary among virus strains [3,25]. We similarly found that these wild bird viruses have different pathotypes in ducks and half of the viruses we tested killed less than 20% of the infected ducks. It is reasonable to speculate that these viruses may not be able to cause disease or death in field adult ducks, which are much more tolerant to influenza viruses than young ducks commonly used in laboratory studies. Thus, our study emphasizes the importance of avian influenza surveillance in domestic ducks.
